# Epigenetic alterations differ in phenotypically distinct human neuroblastoma cell lines

**DOI:** 10.1186/1471-2407-10-286

**Published:** 2010-06-14

**Authors:** Qiwei Yang, Yufeng Tian, Kelly R Ostler, Alexandre Chlenski, Lisa J Guerrero, Helen R Salwen, Lucy A Godley, Susan L Cohn

**Affiliations:** 1 Department of Pediatrics, University of Chicago, 900 Ease 57th Street, KCBD Rm. 5240, Chicago, IL 60637, USA; 2 Department of Medicine, University of Chicago, 900 Ease 57th Street, KCBD Rm. 7230, Chicago, IL 60637, USA; 3 Department of Medicine, University of Chicago, 900 Ease 57th Street, KCBD Rm. 7124, Chicago, IL 60637, USA; 4 Department of Pediatrics, University of Chicago, 900 Ease 57th Street, KCBD Rm. 5100, Chicago, IL 60637, USA

## Abstract

**Background:**

Epigenetic aberrations and a CpG island methylator phenotype have been shown to be associated with poor outcomes in children with neuroblastoma (NB). Seven cancer related genes (*THBS-1, CASP8, HIN-1, TIG-1, BLU, SPARC*, and *HIC-1*) that have been shown to have epigenetic changes in adult cancers and play important roles in the regulation of angiogenesis, tumor growth, and apoptosis were analyzed to investigate the role epigenetic alterations play in determining NB phenotype.

**Methods:**

Two NB cell lines (tumorigenic LA1-55n and non-tumorigenic LA1-5s) that differ in their ability to form colonies in soft agar and tumors in nude mice were used. Quantitative RNA expression analyses were performed on seven genes in LA1-5s, LA1-55n and 5-Aza-dC treated LA1-55n NB cell lines. The methylation status around *THBS-1, HIN-1, TIG-1 *and *CASP8 *promoters was examined using methylation specific PCR. Chromatin immunoprecipitation assay was used to examine histone modifications along the *THBS-1 *promoter. Luciferase assay was used to determine *THBS-1 *promoter activity. Cell proliferation assay was used to examine the effect of 5-Aza-dC on NB cell growth. The soft agar assay was used to determine the tumorigenicity.

**Results:**

Promoter methylation values for *THBS-1*, *HIN-1*, *TIG-1*, and *CASP8 *were higher in LA1-55n cells compared to LA1-5s cells. Consistent with the promoter methylation status, lower levels of gene expression were detected in the LA1-55n cells. Histone marks associated with repressive chromatin states (H3K9Me3, H3K27Me3, and H3K4Me3) were identified in the *THBS-1 *promoter region in the LA1-55n cells, but not the LA1-5s cells. In contrast, the three histone codes associated with an active chromatin state (acetyl H3, acetyl H4, and H3K4Me3) were present in the *THBS-1 *promoter region in LA1-5s cells, but not the LA1-55n cells, suggesting that an accessible chromatin structure is important for *THBS-1 *expression. We also show that 5-Aza-dC treatment of LA1-55n cells alters the DNA methylation status and the histone code in the *THBS-1 *promoter modifies cell morphology, and inhibits their ability to form colonies in soft agar.

**Conclusion:**

Our results suggest that epigenetic aberrations contribute to NB phenotype, and that tumorigenic properties can be inhibited by reversing the epigenetic changes with 5-Aza-dC.

## Background

The pediatric cancer neuroblastoma (NB) is characterized by a broad spectrum of clinical behavior, reflective of the biologic heterogeneity of this neoplasm [1,2]. Although specific genetic abnormalities have been shown to be predictive of the outcome in NB, recent studies have indicated that epigenetic aberrations also contribute to NB pathogenesis. Our group and others have shown that poor outcome is associated with hypermethylation of a number of tumor suppressor genes including *RASSF1A*, *CASP8*, *HIN-1*, and *DCR2 *[3-5]. We have also demonstrated that NB tumor growth is impaired following treatment with drugs that inhibit histone deacetylase and/or DNA methylation in preclinical models [6].

Biologic differences are commonly seen in NB cell lines established from human NB tumors, and morphologically distinct cell types [neuroblastic (N-type) and substrate-adherent (S-type)] have been extensively characterized. N-type cells have small, rounded, loosely adherent cell bodies with numerous neurite-like processes and express neuronal markers, such as tyrosine hydroxylase. S-type cells are substrate-adherent large, flat cells that resemble epithelial cells or fibroblasts and lack neuronal markers [7,8]. Interestingly, purified N-type and S-type NB cells can spontaneously interconvert from one cell type to the other, suggesting that epigenetic changes that are reversible may play a role in determining phenotype.

A CpG island methylator phenotype (CIMP) has been shown to be predictive of poor outcome in a variety of different cancers, including NB [9]. In studies by Abe and coworkers, CIMP was detected in almost all *MYCN*-amplified NB tumors, a genetic marker of poor prognosis. However, CIMP was also shown to be predictive of poor outcome in cases without *MYCN *amplification [10-12]. Further, in this study the prognostic influence of CIMP and *MYCN*-amplification was independent of age and disease stage, both powerful clinical prognostic factors in NB.

To further investigate the role epigenetic changes play in determining NB phenotype, we analyzed the status of promoter methylation and the level of expression of seven genes with tumor suppressor function (*THBS-1, HIC-1, HIN-1 TIG-1, CASP8, BLU*, and *SPARC*), that are epigenetically silenced in subsets of adult cancers, in an N-type tumorigenic NB cell line (LA1-55n) and an S-type non-tumorigenic NB cell line (LA1-5s). Changes in NB cell morphology and soft agar colony growth following pharmacological reversal of the epigenetic aberrations were also examined.

## Methods

### Cells and culture conditions

The biological and genetic characteristics of the NB cell lines used in this study have been previously reported [13]. NB cell lines were grown at 5% CO_2 _in RPMI 1640 (Invitrogen, Carlsbad, CA) supplemented with 10% heat-inactivated fetal bovine serum (Invitrogen), L-Glutamine and antibiotics.

### 5-Aza-dC and VPA treatment

Cells were treated with either the DNA methyltransferase inhibitor 5-Aza-dC or the histone deacetylase inhibitor VPA as shown in Table 1.

**Table 1:** Conditions for 5-Aza-dC and VPA treatments

5-Aza-dC treatment		
Experiment	5-Aza-dC (μM)	time (days)
cell proliferation	0.01, 0.1, 1, 10, 100	3
gene expression	4	1
methylation study	4	1
ChIP assay	4	1
soft agar assay	0.5, 2.5, 10	2
soft agar assay	0.1, 1	7
morphology	0.1	14, 21
		
VPA treatment		
Experiment	VPA (mM)	time (days)
ChIP assay	5	1

### cDNA synthesis and SYBR green real-time PCR

RNA was isolated from untreated and 5-Aza-dC-treated LA1-55n and LA1-5s cells using Trizol reagent (Invitrogen). Reverse transcription was performed using *Superscript *III (Invitrogen) and 50 μM oligo(dT)_20 _at 50°C for 50 min. SYBR green real-time PCR reactions were set up containing 1X Power SYBR Green Master Mix (Applied Biosystems, Foster City, CA), 250 nM forward and reverse primers in a 20 μl reaction. All assays were carried out in a 96-well format. Real-time fluorescent detection of PCR products was performed with an 7500 Fast Real-Time PCR System (Applied Biosystems) using the following thermocycling conditions: 1 cycle of 50°C for 2 min and 95°C for 20 s; 40 cycles of 95°C for 30 s, and 60°C for 1 min. The primers and PCR conditions are shown in Table 2. *GAPDH *was used as an endogenous control for gene expression. For data analysis, the comparative method (∇∇Ct) was used to calculate relative quantities of a nucleic acid sequence. Non-treated LA1-55n cells were used as the calibrator sample, and *GAPDH *was used as an endogenous control detector.

**Table 2:** Primer sequences for PCR

Gene	Purpose	Sense primer	Antisense primer
*BLU*	Q-PCR	CACGAGGCCTCCATCATCA	ACTCACACACCTCCTTGTGGAA
*CASP8*	Q-PCR	TGCAGAGGGAACCTGGTACAT	TCGAGGACATCGCTCTCTCA
*GAPDH*	Q-PCR	CCATGGGGAAGGTGAAGGTCGGACTC	GGTGGTGCAGGCATTGCTGATG
*HIC-1*	Q-PCR	TGCTGCAGCTCAACAACCA	GGCGTTCTGCACCACGAT
*HIN-1*	Q-PCR	CACCCTCAACCCGCTGAA	ACACTTCTGGGAGCCCTCTATG
*SPARC*	Q-PCR	TCTTCCCTGTACACTGGCAGTTC	AGCTCGGTGTGGGAGAGGTA
*THBS1*	Q-PCR	TGGAACTATGGGCTTGAGAAAAC	CACTGATGCAAGCACAGAAAAGA
*TIG-1*	Q-PCR	CCTGGCAAACCTCTTAAAGTGAA	GGAGGCTTCTTCTGGTGTCTGT
			
*Actin*	QMSP	TGGTGATGGAGGAGGTTTAGTAAGT	AACCAATAAAACCTACTCCTCCCTTAA
*BLU*	QMSP	GTAGTTATGGAAACGGGTTAGTC	CGCTAAAAATCCAAATACTATAACG
*CASP8*	QMSP	AGGTAGGAGAATCGTTTGAATTC	AACGAAATTTCGCTCTTATTACC
*HIC-1*	QMSP	GTTAGGCGGTTAGGGCGTC	CCGAACGCCTCCATCGTAT
*HIN-1*	QMSP	AGGGACGATTAGGTTTTATTTTC	CTAATTTCGAAAACAAAAAAACG
*MYOD1*	QMSP	CCAACTCCAAATCCCCTCTCTAT	TGATTAATTTAGATTGGGTTTAGAGAAGGA
*SPARC*	QMSP	GTTGTTATATTCGGGGACGAC	TAACCCGTTTCCATAACTACGA
*THBS1*	QMSP	GAAAGGGTTCGAAGGTAGC	CCGAACGCCTATCCTAAA
*TIG-1*	QMSP	GTTTAGTGTTAGGATGCGGTATC	AAAACGCGATACGAAATAACA
			
*THBS1*	ChIP-1	ATGACAACTTGGCAAAAAAGAGAA	GGCCGAAGTGATGGACCTT
*THBS1*	ChIP-2	AATGTGGGTGAATTCCTGTTAAGG	GGGTGGAAAGGAAAGGTCATAGA
*THBS1*	ChIP-3	TGGGTGCCGATTCCAGAA	TCCCGCAAATCATAGGTAATGA
*THBS1*	ChIP-4	CCACGCAAGAAAAGCGAAA	TGCTGCAAACAGCCAAGTG
*THBS1*	ChIP-5	GGGCAGGTACTTTAACGAATGG	CTGGGCCCGTTTTGTAAAAA
*THBS1*	ChIP-6	GGCGGGCACCGACTTT	GCGCAACTTTCCAGCTAGAAA

### DNA isolation and bisulfite modification

Total genomic DNA was extracted from the NB cell lines using the Puregene Core Kit A (Qiagen, Valencia, CA) and modified by sodium bisulfite using the CpGenome DNA Modification Kit (Intergen Co., Purchase, NY). Genomic DNA from normal human adrenal tissue was purchased from BioChain Institute, Inc. (Hayward, CA). As previously described [13], 1 μg of genomic DNA was denatured by NaOH and modified by sodium bisulfite, which converts all unmethylated cytosines to uracils. The modified DNA was desulfonated with NaOH and purified.

### DNA methylation analysis

DNA methylation degree was measured using real-time quantitative methylation-specific PCR (QMSP). The real-time PCR conditions were 95°C for 10 minutes, then 40 cycles at 95°C for 15 seconds, and 58°C to 60°C (depending on the primer set and/or optimization conditions) for 1 minute. Fluorescence data was collected during the annealing/extension step for determining the cycle threshold (Ct). After amplification, melting curve analysis was performed for PCR product identification that consisted of one cycle of 95°C for 1 minute, 58-60°C for 30 seconds, and 95°C for 30 seconds, with data collection throughout the linear increase of temperature from 58 to 95°C. Serial dilutions of the LA1-55n DNA template were used to generate standard curves from Ct values to assess the sensitivity, amplification efficiency, and linear range for quantification. All real-time QMSP assays were performed in duplicate or triplicate, and at least three independent experiments were performed for each condition tested. The primer sequences and conditions are shown in Table 2. Both *β-actin *and *MYOD1 *were used as internal reference genes. Primers of the *β-actin *and *MYOD1*genes were located in an area without CpG nucleotides; thus, amplification of *β-actin *and *MYOD1 *by QMSP occurs independently of a CpG island methylation status, whereas the amplification of *THBS1, HIN1, TIG1*, and *CASP8 *is proportional to the degree of cytosine methylation within their promoters.

### Chromatin immunoprecipitation (ChIP) assay

NB cells (1 × 10^7^) were treated with 1% formaldehyde for 10 min to crosslink histones to DNA. After washing with cold PBS, the cell pellets were resuspended in a cell lysis buffer (10 mM Tris, pH8.0, 10 mM NaCl, 0.2% NP40). Nuclei were resuspended in nuclei lysis buffer (50 mM Tris pH 8.0, 10 mM EDTA, 1% SDS) and sonicated 15 min for LA1-55n and 20 min for LA1-5s cells. The soluble chromatin fraction was collected, and 5 μl of antibody for acetyl-H3 (Upstate, Waltham, MA), acetyl-H4 (Upstate), trimethyl-H3-K4 (Abcam, Cambridge, MA), trimethyl-H3-K9 (Upstate), trimethyl-H3-K27 (Upstate), dimethyl-H3-K27 (Abcam), or normal rabbit IgG was added. After incubation, chromatin-antibody complexes were collected using salmon sperm DNA/protein A agarose beads (Upstate). After washing, immunoprecipitated DNA was treated with RNase and the crosslinks were then reversed by heating the samples at 65°C for 6 h. DNA was extracted with a QIAquick PCR Purification kit (Qiagen) and analyzed by SYBR green real-time PCR. The primer pairs used for ChIP assays are also shown in Table 2.

### Luciferase assays

A series of *THBS-1 *reporter plasmids [13] were used to determine the *THBS-1 *promoter activity. LA1-55n and LA1-5s cells were seeded in a 24-well dish and grown to 90% confluence in the growth medium. For each well, 0.5 μg of reporter construct was cotransfected with 25 ng of the Renilla luciferase plasmid, pRL-CMV (Promega, Madison, WI), into NB cells using Lipofectamine2000 (Invitrogen). Cells were harvested 36 h after transfection, and the reporter activity for the two cell lines was measured at the same time using the Dual Luciferase Assay (Promega) according to the manufacturer's instructions. Experimental luciferase activities were normalized for efficiency against control Renilla luciferase readings.

### Cell proliferation, examination of morphological alteration, and soft agar clonogenic assay

MTS colorimetric assays were performed to measure cell proliferation. Briefly, LA1-55n cells were seeded into 96-well plates at a density of 5 × 10^3 ^cells/well. After 24 h, 5-Aza-dC was added at various concentrations to each quadruplicate well. After 72 h treatment, MTS labeling mixture (Promega) was added, and cells were further incubated for 3 h. The absorbance of the samples was measured using a Bio-Kinetics Microplate Reader (Bio-Tek Instruments, Winooski, VT). Percentage of N-type cells with or without treatment with 5-Aza-dC was obtained by quantifying 5 consecutive high power fields. Anchorage-independent growth was assayed by colony formation on soft agar. Briefly, approximately 2 × 10^3 ^cells from the 5-Aza-dC treated and nontreated LA1-55n cells and LA1-5s cells were plated on an upper layer of 0.3% Bacto-Agar in RPMI 1640 and 20% fetal bovine serum over an underlayer of 0.51% Bacto-Agar with 20% fetal bovine serum, RPMI 1640, and antibiotics in a 35-mm dish as described previously [14]. Colonies were counted on day 28.

### Statistical analyses

Statistical analyses were performed using a two-tailed Student's *t *test. A p value of ≤ 0.05 was considered statistically significant.

## Results

### Expression analysis of cancer related genes

**Figure 1:**
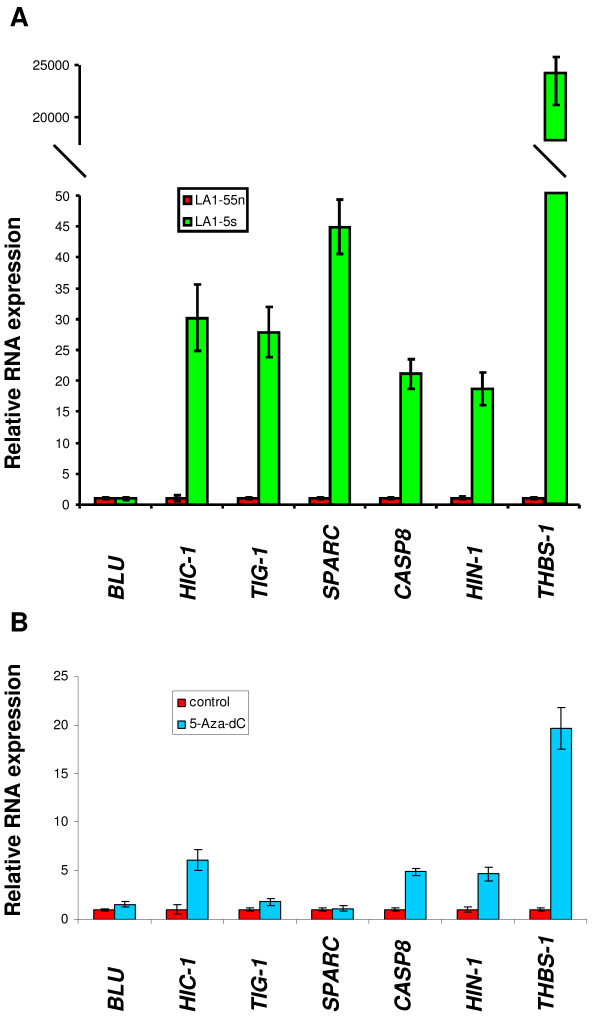
**Cancer related gene expression in tumorigenic, non-tumorigenic, and 5Aza-dC treated tumorigenic NB cell lines**. A, Quantitative RT-PCR analysis of RNA expression of seven cancer related genes was performed in tumorigenic LA1-55n and non-tumorigenic LA1-5s NB cell lines. *GAPDH *was used as an endogenous control. B, LA1-55n cells were treated with 4 μM 5-Aza-dC for 1 day. Quantitative RT-PCR analysis of seven cancer related genes was performed in tumorigenic LA1-55n treated with 5-Aza-dC. *GAPDH *was used as an endogenous control.

To investigate the relationship between epigenetic changes and NB phenotype, we analyzed the level of expression of seven genes with tumor suppressor function that are epigenetically silenced in a variety of adult and pediatric cancers (*THBS-1, HIC-1, HIN-1 TIG-1, CASP8, BLU*, and *SPARC*), in a tumorigenic N-type NB cell line (LA1-55n) and a non-tumorigenic S-type cell line (LA1-5s). Lower levels of *THBS-1*, *SPARC*, *HIC-1*, *HIN-1*, *TIG-1 *and *CASP8 *expression were detected in the tumorigenic LA1-55n cells compared to non-tumorigenic LA1-5s cells (Figure 1A). No difference in the level of *BLU *gene expression was seen.

### Treatment with 5-Aza-dC restores gene expression

To test if the decreased levels of gene expression detected in the tumorigenic LA1-55n cells were due to promoter hypermethylation, the level of gene expression was examined in LA1-55n cells following treatment with 5-Aza-dC, a DNA-methyltransferase inhibitor. As shown in Figure 1B, the level of expression of five of the genes (*HIN-1*, *CASP8*, *HIC-1*, *THBS-1*, and *TIG-1*) was upregulated with 4 μM of 5-Aza-dC treatment at 24 h. This dose and time point has previously been shown to restore gene expression in NB cells [13]. *THBS-1 *was especially sensitive to 5-Aza-dC treatment, and the level of transcript expression was increased by 20 folds following 24 h of treatment. However, no significant upregulation of *BLU *and *SPARC *was seen following 24 h of 5-Aza-dC treatment.

### DNA methylation profiling of cytosine methylation

**Figure 2:**
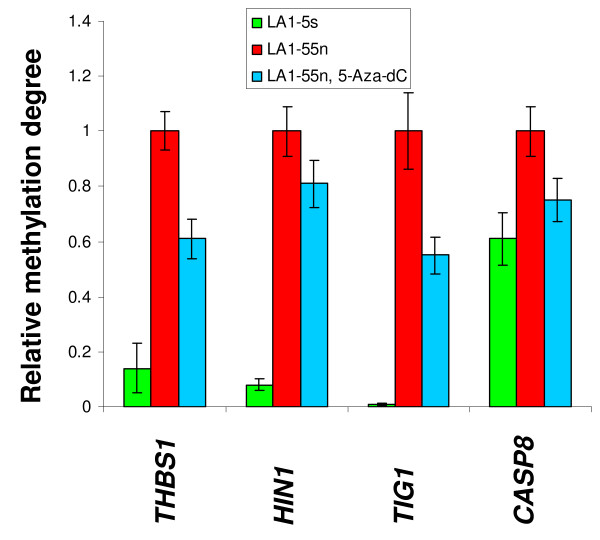
**Alteration of DNA methylation degree after treatment with 5-Aza-dC**. Genomic DNA from LA1-55n, LA1-5s, and LA1-55n treated with 4 μM 5-Aza-dC was isolated and modified by sodium bisulfite. Quantitative methylation-specific PCR was performed to examine the methylation degree around promoter regions for four cancer related genes (*THBS-1, HIN-1, TIG-1*, and *CASP8*) in LA1-55n, LA1-5s as well as LA1-55n cells treated with 5-Aza-dC.

To examine the status of promoter DNA methylation of these genes in the tumorigenic LA1-55n and non-tumorigenic LA1-5s NB cells, quantitative methylation analysis was performed for the promoter regions of four genes. As shown in Figure 2, we found a higher degree of DNA methylation within the *THBS-1*, *CASP8*, *HIN-1*, and *TIG-1 *promoters in tumorigenic LA1-55n cells versus the non-tumorigenic LA1-5s cells. In addition, the methylation degree for the promoter regions of four genes was decreased after treatment with 5-Aza-dC.

### *THBS-1 *promoter histone acetylation and methylation

**Figure 3:**
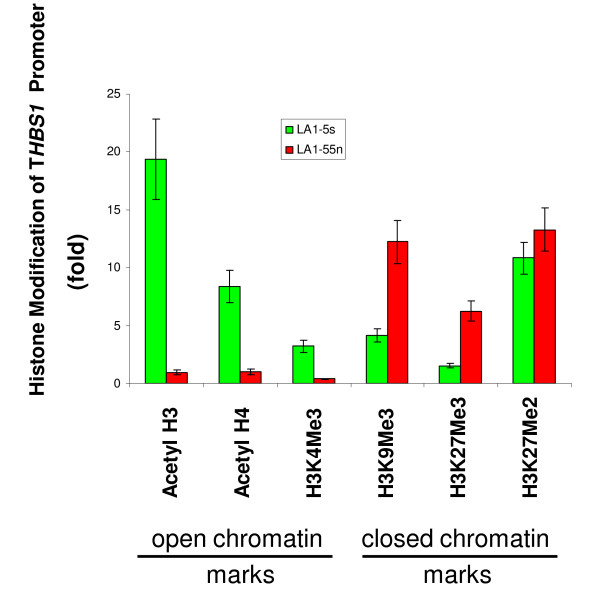
**ChIP assays of the *THBS-1 *CpG islands**. DNA was immunoprecipitated with antibodies specific for acetyl H3, acetyl H4, H3K4Me3, H3K9Me3, H3K27Me3, and H3K27Me2 respectively. Q-PCR analysis of ChIP assay on LA1-55n and LA1-5s cells were performed in *THBS-1 *promoter region.

To investigate if histone modifications contributed to the significant differences in *THBS-1 *expression levels in the tumorigenic versus non-tumorigenic NB cells, we analyzed histone marks along the *THBS-1 *promoter. As shown in Figure 3, acetylated H3 (acetyl H3), acetylated H4 (acetyl H4), and H3K4Me3 marks associated with an open chromatin state, are enriched in the *THBS-1 *promoter regions in the non-tumorigenic LA1-5s cells that express high levels of this gene. However, consistent with the low levels of *THBS-1 *expression in the tumorigenic NB cells, an enrichment of marks associated with a closed chromatin state (H3K9Me3, H3K27Me3, and H3K27Me2) are present in *THBS-1 *promoter in the LA1-55n cells.

**Figure 4:**
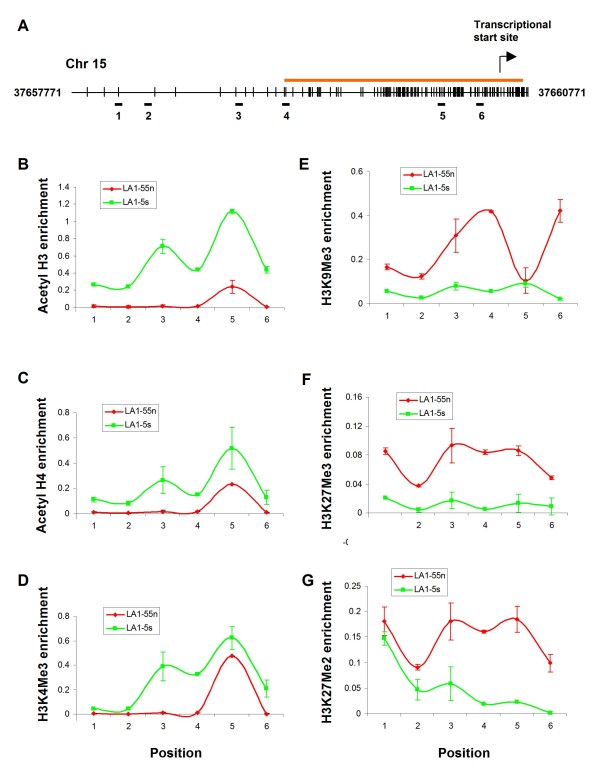
**Histone code map of *THBS-1 *promoter region**. ChIP assay was performed on tumorigenic LA1-55n and non-tumorigenic LA1-5s cells. A, Schematic of the *THBS-1 *promoter. The vertical lines represent the location of CpG dinucleotide, and the orange line indicates the CpG island of the *THBS-1 *promoter region. The horizontal bars below the schematic indicate the location of the DNA fragments amplified by Q-PCR done on DNA recovered from ChIP experiments. B, C, and D, The enrichment of *THBS-1 *promoter DNA immunoprecipitated with antibodies specific for acetylated H3, acetyl H4, and H3K4Me3, respectively in LA1-5s cells. E, F, and G, The enrichment of *THBS-1 *promoter DNA immunoprecipitated with antibodies specific for H3K9Me3, H3K27Me3, and H3K27Me2, respectively in LA1-55n cells.

We also performed detailed mapping of histone acetylation and histone methylation across a 1.2 kb region of the *THBS-1 *promoter in the tumorigenic LA1-55n and non-tumorigenic LA1-5s NB cell lines using the ChIP assay (Figure 4A). Overall, acetylated histone H3 and H4 were enriched throughout the unmethylated *THBS-1 *promoter in the non-tumorigenic LA1-5s cells. In contrast, there was no acetylation of these sites along the hypermethylated promoter in the tumorigenic LA1-55n cells (Figure 4B and 4C). A similar pattern of acetylation in the *THBS-1 *promoter in the NB cells lines was seen for H3K4Me3 (Figure 4D). In contrast, the three histone codes associated with a repressive chromatin state (H3K9Me3, H3K27Me3, H3K27Me3) were enriched along the entire hypermethylated *THBS-1 *promoter in tumorigenic LA1-55n cells (Figure 4E, F, G).

### Treatment with 5-Aza-dC induces histone modifications in the *THBS-1 *promoter

**Figure 5:**
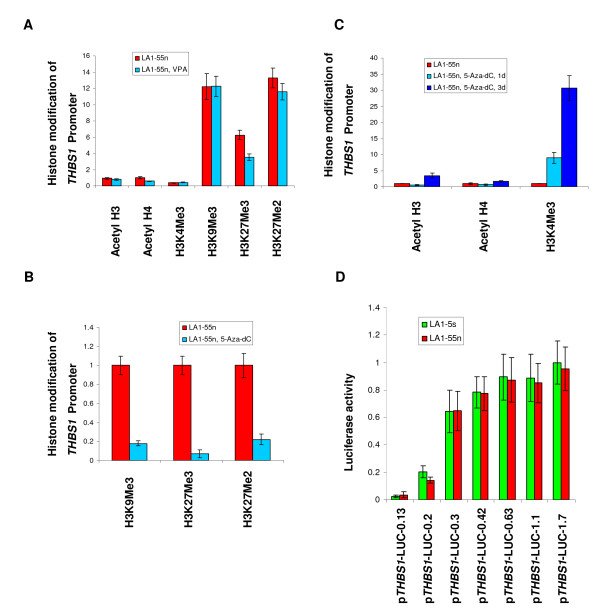
**Alteration of histone marks around *THBS-1 *promoter region**. A, ChIP/quantitative PCR was performed around *THBS-1 *promoter region in LA1-55n and VPA-treated LA1-55n cells. B, ChIP/quantitative PCR was performed around *THBS-1 *promoter region in 5-Aza-dC treated and non-treated LA1-55n cells. The enrichment of three histone marks related to repressive chromatin state is markedly reduced in 5-Aza-dC treated LA1-55n cells compared with untreated ones. C, ChIP assay was performed around *THBS-1 *promoter region in 5-Aza-dC treated and non-treated LA1-55n cells. D, Luciferase assay was performed to examine the promoter activity of *THBS-1 *between LA1-55n and LA1-5s cells.

In a previous study, we showed that administration of the HDAC inhibitor valproic acid (VPA) changed gene expression in NB cells [6]. The cells were treated with 1 mM VPA for 2-48 h. Based on these results, we treated the tumorigenic LA1-55n cells with 5mM VPA for 1 day and investigated its effects on histone modifications. Unexpectantly, the ChIP assays revealed that VPA treatment alone did not induce an enrichment of markers associated with open chromatin state along the *THBS-1 *promoter region. Furthermore, VPA treatment did not decrease an enrichment of marks associated with closed chromatin state, except for H3K27Me3 (Figure 5A).

To determine the relation between DNA methylation and histone modification, we next tested the effects of 5-Aza-dC, an inhibitor of DNMT, on the histone marks, and found that this DNA methyltransferase inhibitor did induce histone modifications. Following treatment of the tumorigenic LA1-55n cells with 5-Aza-dC, the levels of H3K9Me3, H3K27Me3, and H3K27Me2 were severely depleted (Figure 5B). Whereas the level of acetylated H3K4Me3,acetyl H3, and acetyl H4 along the *THBS-1 *promoter region was markedly enriched after 3 d treatment with 5-Aza-dC (Figure 5C). After 24 h of 5-Aza-dC-treatment, only the enrichment of H3K4Me3 is observed.

### *THBS-1 *promoter activity

To investigate if *THBS-1 *promoter activity contributed to the difference of *THBS-1 *expression in the tumorigenic LA1-55n versus non-tumorigenic LA1-5s cells, a series of *THBS-1 *luciferase/promoter reporter constructs were used and transiently transfected into the phenotypically distinct NB cell lines (LA1-55n and LA1-5s). As shown in Figure 5D, no significant difference in the activity of the *THBS-1 *promoter was seen in the cell lines, indicating that transcriptional factors are functional in both NB cell lines. Thus, the disparate levels of *THBS-1 *expression in the LA1-55n and LA1-5s cells are not due to differences in promoter activity of *THBS-1 *in the tumorigenic LA1-55n and non-tumorigenic LA1-5s cells.

### 5-Aza-dC treatment modifies the tumorigenic phenotype of LA1-55n NB cells

**Figure 6:**
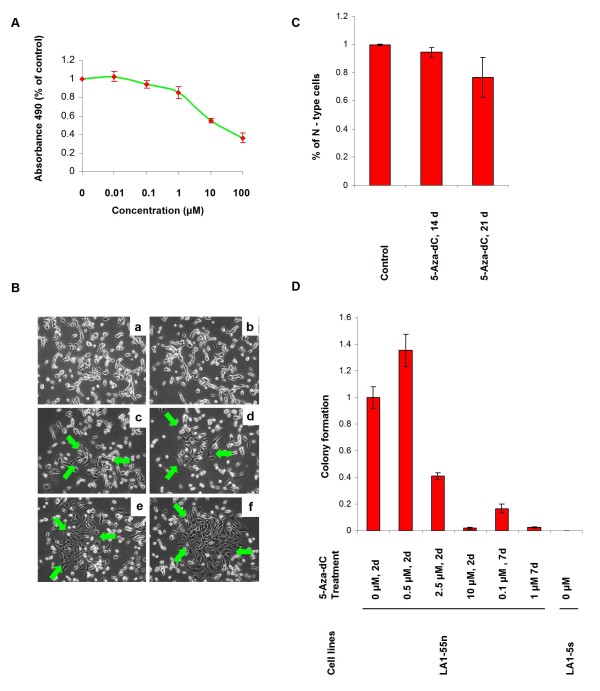
**Effect of 5-Aza-dC on NB growth and tumorigenicity *in vitro***. A, *In vitro *cell proliferation assay demonstrating dose-response effects of 5-Aza-dC on LA1-55n cells. Each point represents the mean of four replicate experiments. B, Morphological alteration after treatment with 5-Aza-dC. LA1-55n cells were treated with 0.1 μM of 5-Aza-dC for 14 and 21 days respectively. Top photos (a and b) show the morphology of nontreated LA1-55n cells. Middle photos (c and d) show the morphology of LA1-55n cells treated with 5-Aza-dC for 14 days. Bottom photos (e and f) show the morphology of LA1-55n cells treated with 5-Aza-dC for 21 days. Green arrows indicated the flat "S" type cells. C, Quantitative analysis of percentage of N- type cells after treatment with 5-Aza-dC for 14 and 21 days. D, Quantitative analysis of colony formation on soft agar for 5-Aza-dC treated and untreated NB cells. Colonies were counted under a microscope. The average number of colonies was calculated and is shown as a percentage of the average number of colonies in the untreated group per cell line. Error bars represent standard deviation of colony numbers between different plates for each group.

To investigate if reversal of the epigenetic aberrations in the tumorigenic LA1-55n cells with 5-Aza-dC treatment was sufficient to induce changes in phenotype, we first examined its effects on cell proliferation. We found that the treatment inhibited the proliferation of LA1-55n NB cells *in vitro *in a dose-dependent manner, with an ID_50 _of 10 μM (Figure 6A). We next assessed whether treatment with 5-Aza-dC would induce changes in the morphology of the N-type LA1-55n cells. For these studies, the cells were treated with 0.1 μM 5-Aza-dC, a dose that is not cytotoxic. Following 21 days of 5-Aza-dC treatment, substrate-adherent cells, resembling S-type NB cells, were seen (Figure 6B, as shown with arrows), and the number of cells with neurites decreased by ~20% (p = 0.0062) (Figure 6C). Treatment with 5-Aza-dC also decreased the ability of LA1-55n to form colonies in soft agar in a dose dependent manner (Figure 6D). At a concentration of 10 μM, the ID_50_, the number of colonies was decreased by 95% compared to controls (p < 0.001). The colony formation was markedly decreased after 7 days of treatment even at concentrations of 0.1 μM (p < 0.001). As expected, the non-tumorigenic NB cell line, LA1-5s did not form colonies on the soft agar.

## Discussion

Epigenetic changes play an important role in the pathogenesis of cancer, and a CpG island methylator phenotype has been shown to be predictive of poor outcome. To investigate if epigenetic aberrations contribute to NB phenotype, we examined the methylation status and level of expression of seven genes with known tumor suppressor function (*HIC-1, TIG-1, HIN-1, CASP8, THBS-1, SPARC*, and *BLU*) in a N-type tumorigenic NB cell line (LA1-55n) and an S-type, non-tumorigenic NB cell line (LA1-5s). We show that the tumorigenic LA1-55n cells have higher levels of promoter methylation and lower levels of expression of five of the genes (*THBS-1*, *HIC-1*, *HIN-1*, *TIG-1*, and *CASP8*) compared to the non-tumorigenic LA1-5s cells. We also show that cell morphology and phenotype of the tumorigenic LA1-55n cells are modified following treatment with the demethylating agent 5-Aza-dC.

Each of the genes analyzed in this study has previously been shown to be epigenetically silenced in other types of cancer, and in some cases, the presence of abnormal promoter methylation has been shown to be associated with a more aggressive phenotype in adult or pediatric cancers. *SPARC *is an extracellular matrix protein that has been shown to function as an inhibitor of angiogenesis [15], and in lung adenocarcinomas SPARC methylation has been correlated with a negative prognosis [16]. Athough disparate levels of SPARC expression was seen in the NB cell lines, there was no evidence of SPARC methylation in the NB cells, indicating that alternative mechanisms regulate SPARC expression in NB. In contrast, we did find higher levels of methylation of the pro-apoptotic gene *CASP8 *in the tumorigenic LA1-55n cells, which is consistent with the association between *CASP8 *methylation and high-risk disease and poor outcome that has been reported in primary NB tumors [4]. *BLU *functions as a tumor supressor in many cancer types [17]. In human nasopharyngeal carcinoma, the frequency of *BLU *promoter methylation was much higher compared with non-neoplastic nasopharyngeal epithelia [18]. However, we found no significant difference in the level of expression of this gene in the NB cell lines.

*HIC-1 *is a newly discovered tumor suppressor and transcriptional repressor which is located at 17p13.3, a region which frequently undergoes allelic loss in human cancers [19]. A recent study indicated that HIC-1 is a central molecule in a novel mechanism controlling cell growth and that the disruption of the HIC-1 mediated pathway may lead to abnormal cell proliferation [20]. Moreover, low to undetected expression of *HIC-1 *is associated with poor outcome in breast cancer [21]. In this study, expression of *HIC-1 *is higher in non-tumorigenic NB cells compared with tumorigenic cells.

*TIG-1*, which has been linked to retinoic acid signaling, was shown to be downregulated in many cancers [22,23]. Forced expression of *TIG1 *in cancer cells results in decreased invasiveness and tumorigenicity, indicating that diminished expression of *TIG1 *is involved in the malignant progression of cancer. Our study showed that lower level expression of *TIG-1 *in tumorigenic NB cells is associated with hypermethylation of its promoter. *HIN-1 *is a putative cytokine with growth inhibitory activities. *HIN-1 *was initially found to be significantly downregulated in human breast carcinomas and in preinvasive lesions. *HIN-1 *is a potent inhibitor of anchorage-dependent and anchorage-independent cell growth, cell migration, and invasion [24,25]. Our previous study indicated that methylation of *HIN-1 *is associated with poor outcome in NB [4]. In this study we showed that the methylation degree of *HIN-1 *promoter region is much higher in tumorigenic NB cells compared with non-tumorigenic NB cells.

Because of the remarkable up-regulation of *THBS-1 *expression that was seen in the LA1-55n cells following treatment with 5-Aza-dC, we performed additional studies examining the histone marks along the promoter regions of this gene in the NB cell lines. Histone marks associated with a repressive chromatin state (H3K9Me3, H3K27Me3, and H3K4Me3) were detected in the tumorigenic LA1-55n cells, whereas histone codes associated with an active chromatin state (acetyl H3, acetyl H4, and H3K4Me3) were present in non-tumorigenic LA1-5s cells. THBS-1 is a well-known natural inhibitor of angiogenesis, and down-regulation of THBS-1 plays a critical role in the angiogenic switch in several tumor types. We have previously shown that the *THBS-1 *promoter is methylated and silenced in tumorigenic NB cell lines and in a cohort of NB primary tumors [13]. In this study, we compiled a detailed map of histone acetylation and methylation across a 1.2 kb region of the *THBS-1 *promoter which indicates that histone acetylation also plays an important role in regulating *THBS-1 *expression. Furthermore, the results of our ChIP assay indicate that an accessible chromatin structure is important in *THBS-1 *expression. To our knowledge, this is the first report showing that *THBS-1 *expression is regulated by histone modification. Our results further suggest that the alteration of key parameters of the histone code depend upon inhibition of the DNMTs, and DNA hypermethylation mediated by DNMTs may be essential for maintaining a particular combination of histone modifications at gene promoters silenced with aberrant DNA methylation.

## Conclusion

Taken together, our results indicate that epigenetic gene silencing contributes to NB phenotype and that by restoring the expression of tumor suppressor genes, 5-Aza-dC can inhibit tumorigenic properties of NB cells. Additional experiments investigating the global changes in gene methylation and expression in control and 5-Aza-dC-treated NB cells are needed to identify the cellular pathways that are modified by this treatment that influence NB phenotype. Further studies investigating the anti-NB effects of agents capable of reversing these epigenetic changes are warranted.

## Abbreviations

The abbreviations used are: *HIC-1*: Hypermethylated in cancer 1; *SPARC*: Secreted protein, acidic and rich in cysteine; *TIG-1*: Tazarotene-induced gene 1; *HIN-1*: High in Normal 1; *THBS-1*: Thrombospondin-1; HDAC: Histone deacetylation; DNMT: DNA methyltransferase.

## Competing interests

The authors declare that they have no competing interests.

## Authors' contributions

QY designed cellular and molecular experiments, performed molecular experiments, and drafted the manuscript. YT and LJG performed cellular experiments. KRO, AC, and HRS participated in the design of the study and revised the manuscript. LAG participated in the design of the study, study coordination, and revised the manuscript critically. SLC participated in the overall design, study coordination and finalized the draft of the manuscript. All authors read and approved the final manuscript.

## Pre-publication history

The pre-publication history for this paper can be accessed here:

http://www.biomedcentral.com/1471-2407/10/286/prepub
